# MR and Ultrasound for Liver Fat Assessment in Children: Techniques and Supporting Evidence

**DOI:** 10.1002/jmri.29756

**Published:** 2025-03-05

**Authors:** Suraj D. Serai, Manish Dhyani, Saubhagya Srivastava, Jonathan R. Dillman

**Affiliations:** ^1^ Department of Radiology Children's Hospital of Philadelphia Philadelphia Pennsylvania USA; ^2^ Perelman School of Medicine University of Pennsylvania Philadelphia Pennsylvania USA; ^3^ Department of Radiology University of Washington Seattle Washington USA; ^4^ Department of Radiology Cincinnati Children's Hospital Medical Center Cincinnati Ohio USA; ^5^ Department of Radiology University of Cincinnati College of Medicine Cincinnati Ohio USA

**Keywords:** diffuse liver diseases, liver fat, PDFF, quantitative MRI, quantitative ultrasound, steatosis

## Abstract

Hepatic steatosis is a common imaging finding that can be a sign of chronic liver disease, most often associated with metabolic dysfunction‐associated steatotic liver disease (MASLD). Imaging techniques for evaluating steatosis range from basic qualitative assessments to advanced and highly accurate quantitative metrics. Among these, MRI‐based proton density fat fraction (PDFF) is widely regarded as a reliable and precise imaging biomarker for quantifying liver steatosis. Additionally, multiple ultrasound platforms now offer quantitative assessments of hepatic steatosis. These methods include attenuation coefficient, speed of sound, backscatter, or other multiparametric approaches such as ultrasound‐derived fat fraction (UDFF) which combines attenuation and backscatter quantification. Newer and upcoming quantitative ultrasound methods include acoustic structure quantification (ASQ) and tissue scatter distribution imaging (TSI). Therefore, ultrasound‐based liver fat measurements could potentially serve as an effective screening tool in certain clinical settings, such as suspected MASLD. In this review, we describe how, why, and when to use MRI‐ and ultrasound‐based fat quantification techniques for assessing liver steatosis in children. We discuss practical strategies for adapting and optimizing these methods in pediatric settings, considering clinical indications, patient preparation, equipment needs, acquisition techniques, potential pitfalls, and confounding factors. Additionally, guidance is provided for interpretation and reporting, along with illustrative case examples.

**Evidence Level:** N/A

**Technical Efficacy:** Stage 5

## 1 Introduction

Hepatic steatosis refers to the abnormal buildup of lipids or triglycerides (TG) within hepatocytes [1]. While hepatic steatosis may be a benign or self‐limiting condition, it is also linked to chronic liver disease, most commonly metabolic dysfunction‐associated steatotic liver disease (MASLD) [2]. Previously known as non‐alcoholic fatty liver disease (NAFLD) and non‐alcoholic steatohepatitis (NASH), MASLD and metabolic dysfunction‐associated steatohepatitis (MASH) are characterized by lipid accumulation with minimal histological progression and an advanced inflammatory stage with variable fibrosis and a higher risk of progression to cirrhosis and related complications, respectively. It is estimated that 6%–26% of MASLD patients have MASH [3]. MASLD is often underdiagnosed in children due to healthcare providers' limited recognition, lack of well‐tolerated and available screening tools, and insufficient awareness of associated complications. Childhood MASLD may be linked to certain genetic risk factors or greater sensitivity to environmental triggers. Adults who develop MASLD in childhood may face a heightened risk of early or more severe complications [4]. Cases of MASLD have been reported in children as young as 2 years old, and MASH‐related cirrhosis has been documented as early as age 8 [5].

Patients with MASLD have a higher overall mortality rate compared to the general population, with cardiovascular complications being the primary cause of death, followed by metabolic and liver‐related conditions [6, 7]. The severity of steatosis in MASLD correlates with an increased cardiovascular risk [8]. Recent studies have shown that steatosis severity is also linked to fibrotic progression in MASLD and improvement in MASH, where a reduction in steatosis severity is associated with better outcomes [9, 10]. The systemic and hepatic conditions associated with MASLD emphasize the need for accurate detection and staging of the disease. It is particularly important to distinguish between physiological and pathological fat accumulation and to monitor treatment responses over time [11]. The histopathological characteristics of MASLD in children can differ from those observed in adults, especially in younger patients. Steatosis may be more pronounced or more prominent in zone 1 hepatocytes, while inflammation and fibrosis often begin to accumulate in the portal tracts. Additionally, ballooning is less commonly seen in children [4].

Historically, liver biopsy was the clinical gold standard for assessing MASLD, providing histological insights not captured by traditional qualitative imaging, such as inflammation and hepatocyte injury specific to MASH [12]. While children's biopsies may exhibit pronounced hepatocellular injury, lobular inflammation, and perisinusoidal fibrosis like adults, there are also distinct patterns unique to children. These patterns are characterized by diffuse, severe macrovesicular hepatocellular steatosis, along with portal inflammation and fibrosis, often without ballooning [13]. The etiopathogenesis, prognosis, and treatment responses may vary for children exhibiting these findings. However, liver biopsy is invasive, associated with risks of significant morbidity and mortality, and prone to sampling errors due to the heterogeneous distribution of steatosis. Steatosis grading via biopsy is also semi‐quantitative, ranging from grade 0 (< 5% hepatocytes) to grade 3 (> 66% hepatocytes) [14]. The relatively small core size of biopsies can lead to sampling errors, particularly given the heterogeneous nature of steatosis [15]. Given these limitations, liver biopsy is a suboptimal tool for screening, monitoring, and research.

In recent years, non‐invasive, quantitative imaging techniques have become widely used in the diagnosis and management of MASLD, offering accurate assessments of key disease markers, especially in pediatric populations [16, 17, 18, 19]. This review explores the use of MRI‐ and ultrasound‐based fat quantification methods for assessing liver steatosis in children, detailing practical strategies for adapting these methods to pediatric care. It covers clinical indications, patient preparation, equipment needs, acquisition techniques, and common pitfalls. Additionally, guidance is provided for interpreting and reporting findings, with illustrative case examples included.

## 2 Non‐Invasive Liver Fat Quantification in Children

Conventional methods for assessing steatosis include ultrasound, CT, and MRI. However, due to the risks associated with ionizing radiation and the availability of safer alternatives, conventional CT should not be used as the primary tool for evaluating hepatic steatosis. In pediatric clinical settings, MRI and ultrasound are preferred for non‐invasive measurement of hepatic steatosis [18, 20, 21]. MRI is increasingly being adopted in standard clinical practice for assessing liver fat fraction, driven by its availability and effectiveness as a quantitative tool [17, 18]. Additionally, many ultrasound platforms now offer quantitative measurements of hepatic steatosis [22]. While MRI has been established as the preferred method for assessing hepatic steatosis, advancements in ultrasound technology now offer improved quantitative measures, making it a potentially viable, lower‐cost screening option [23].

## 3 Liver Fat Quantification by MRI

**FIGURE 1:**
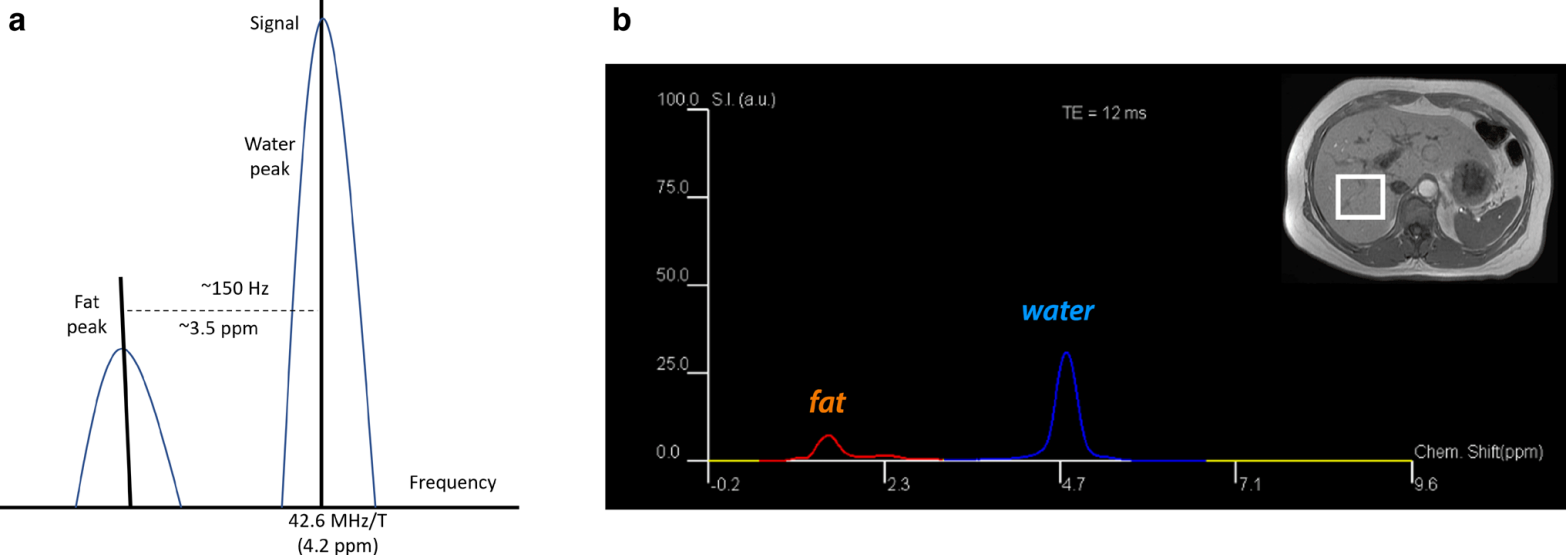
(a) The schematic diagram illustrates the frequency of fat relative to the water frequency. In ^1^HMR spectra, peaks are primarily identified by their frequencies. Fat metabolite frequencies are expressed as shifts relative to a reference, typically the water frequency, in parts per million. (b) MR spectrum obtained in 16‐year‐old boy with Gaucher disease shows the frequency locations of water and lipid peaks. Fat was measured to be 20% in the liver.

MRI has been established as a highly sensitive and specific technique for assessing steatosis [17, 24]. MRI measures the signal intensity of protons based on their resonance frequencies [25]. Fat and water protons resonate at slightly different frequencies due to differences in their chemical structures and molecular electron configurations, allowing for their differentiation (Figure 1a) [26]. Following an applied radiofrequency (RF) pulse, fat protons have a shorter T1 relaxation time and recover longitudinal magnetization more quickly than water protons. These distinct MR properties of fat (i.e., adipose tissue) compared to water protons enable the suppression, identification, and quantification of fat in the liver. MR spectroscopy (MRS) and chemical shift‐based Dixon methods are two popularly used methods to quantify fat using MRI. Both MRS and chemical shift‐based Dixon methods utilize the chemical shift between the resonance frequencies of water and fat. Protons in water molecules experience reduced electronic shielding compared to those in triglycerides, resulting in a resonance frequency for water that is 3.4 ppm higher than that of fat at body temperature. This chemical shift allows both MRS and Dixon‐based MRI methods to differentiate between water and fat proton signals effectively.

## 4 MRI Liver Fat Quantification Methods

### 4.1 MR Spectroscopy

MRS allows for the quantification of metabolites and is considered the most accurate method for measuring hepatic fat content at user‐specified locations within the liver [17, 27, 28]. MRS has proven to be accurate for quantifying fat in vivo, making it a common endpoint in clinical trials for fat‐reducing treatments and medications. With MRS, the proton signals from water and fat are captured from a single voxel during a single breath‐hold (approximately 20 s) and displayed as distinct peaks in a high‐resolution spectrum. The two most commonly used methods for MRS are point‐resolved spectroscopy (PRESS) and stimulated‐echo acquisition mode (STEAM) [26]. While PRESS offers a higher signal‐to‐noise ratio compared to STEAM, the latter is less influenced by J‐coupling that can lead to inconsistency in fat quantification. MRS has been widely used in numerous studies as the reference standard for assessing the accuracy of other methods [29, 30]. To obtain MRS, a volume of about 4 cm^3^ is targeted within the liver (typically in the right liver lobe) (Figure 1b). The MR signal from hydrogen protons in water and from each triglyceride moiety is identifiable by their distinct frequencies, allowing for the quantification of their cumulative signal amplitude [25]. By comparing the cumulative signal amplitude of hydrogen in water versus that in fat, the fat fraction can be determined. Since MRS measures signals from a single voxel, careful voxel placement in a representative, homogeneous region of the liver is crucial to avoid partial volume effects. For instance, the voxel should be positioned away from liver edges, blood vessels, bile ducts, and surrounding visceral fat. However, MRS is limited by sampling bias. Using a small voxel carries the same inherent limitation of biopsy sampling variability. MRS also needs to be managed for physiological motion during data acquisition. Common strategies such as breath‐holding or respiratory navigator triggers need to be selected for accurate liver fat quantification.

### 4.2 MRI Dixon‐Based Method

**FIGURE 2:**
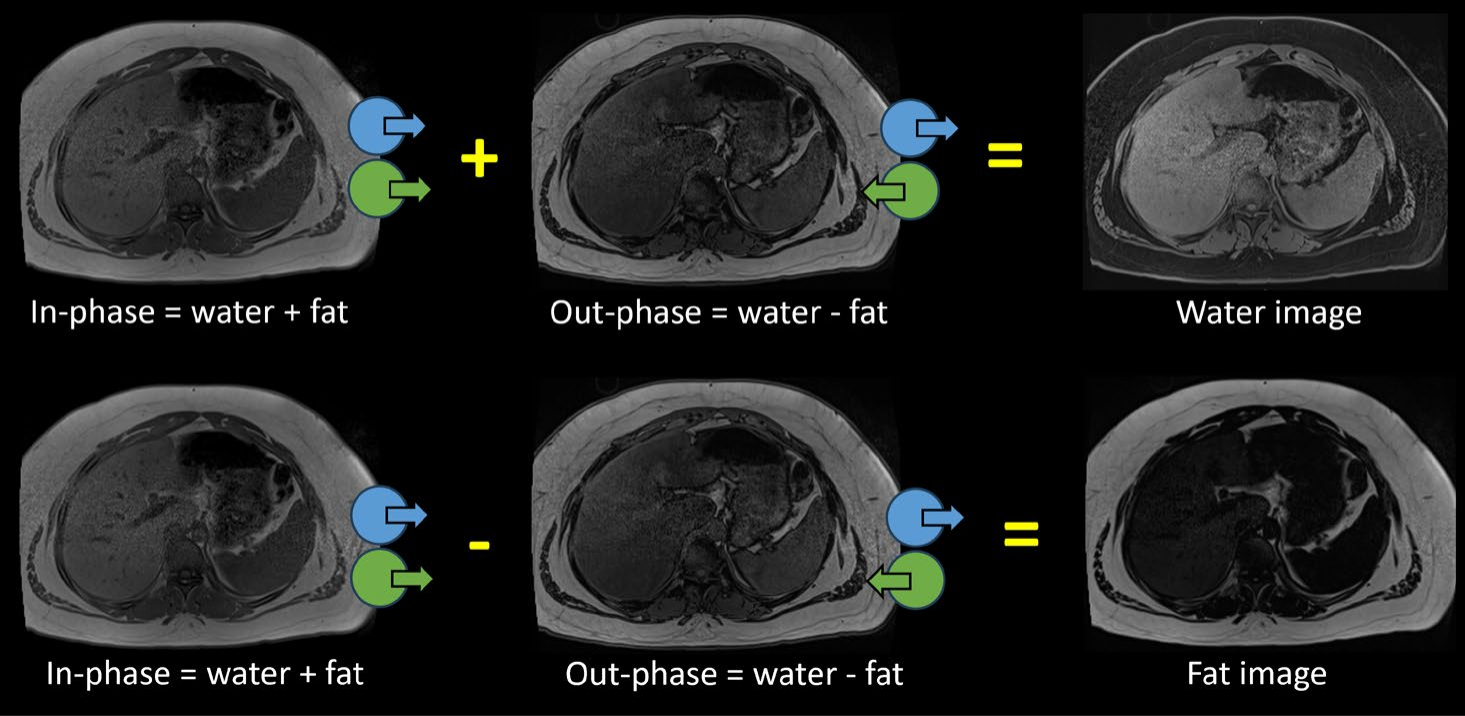
Basics of the Dixon method. The Dixon technique takes advantage of water (blue) and fat (green) precessing at different frequencies by acquiring one image with the water and fat signals in‐phase and another with the signals 180° out‐of‐phase. The images can then be combined mathematically to generate water‐only and fat‐only images.

The MRI Dixon method, also known as chemical‐shift encoded MRI (CSE‐MRI), utilizes the difference in proton resonance frequencies between water and fat. In its simplest form, it achieves this by acquiring images at echo times when water and fat are either in phase or out of phase (Figure 2). The difference in resonance frequencies between water and fat, known as the chemical shift, increases with higher magnetic field strength. This difference enables the differentiation of tissues containing only water from those containing both water and fat. By capturing images at echo times where water and fat signals are approximately in phase (W + F) and outofphase (W‐F), it becomes possible to detect volumetric liver fat by assessing the relative signal loss in the out‐of‐phase images. The chemical shift method (also called two‐point Dixon), initially developed by W. T. Dixon and later refined by others, is often referred to as “Dixon” in the context of MRI techniques for fat‐water separation [31, 32]. Due to its simplicity in calculation, the fat‐water separation can be easily obtained; however, the two‐point Dixon method assumes a zero‐phase shift, and therefore, it fails in regions with large B0 inhomogeneity. In addition, fat has multiple peaks, and the two‐point Dixon method assumes a single peak, leading to underestimation of the fat content.

MRI assessment of hepatic steatosis has progressed from early qualitative methods, such as dual‐echo (two‐point Dixon) chemical shift imaging, to more advanced and fully quantitative approaches aimed at accurate and precise measurement of steatosis. A multi‐peak fat spectrum modeled to a priori calibrated MRS signal is routinely used. Essential advancements include addressing key confounders like R2* decay, the complexity of the fat spectrum (multiple peaks), accounting for the phase shift, and T1 weighting due to the differing T1 values of fat and water [33]. Confounder‐corrected chemical shift‐encoded MRI addresses these issues by using multiple echo times with a low flip angle to minimize T1 weighting and by incorporating the multi‐peak structure of fat into the analysis algorithm [34]. This results in the proton density fat fraction (PDFF), calculated as the ratio of liver fat signal to total signal. The PDFF imaging primarily reflects protons in fat, which constitutes nearly all of the pathological fat in hepatic steatosis [35]. To measure PDFF, a single large region of interest (ROI) that encompasses most of the left and right hepatic lobes can be used. Alternatively, 3–4 circular ROIs (≥ 2 cm) can be placed across the liver on PDFF maps. The idea is to sample a representative area of hepatic parenchyma while avoiding large vessels, focal lesions, artifacts, and the liver edge. The mean PDFF value is reported as a percentage to the nearest whole number, with the range included if steatosis is heterogeneous. ROIs can be placed across multiple slices to ensure comprehensive liver sampling. Multiple studies have shown excellent reproducibility of MRI‐PDFF across varying field strengths, sites, vendors, and protocols [18].

Studies in children with known or suspected NAFLD have demonstrated that MRI‐PDFF offers high intra‐ and inter‐exam repeatability across different scanners and magnetic field strengths [36]. MRI‐PDFF strongly correlates with biochemically determined fat concentrations and MR spectroscopy, showing high precision and accuracy in detecting even small changes in fat fraction, as low as 1.6% over time [37].

### 4.3 Technical Considerations in MRI‐Based Fat Quantification in Children

**FIGURE 3:**
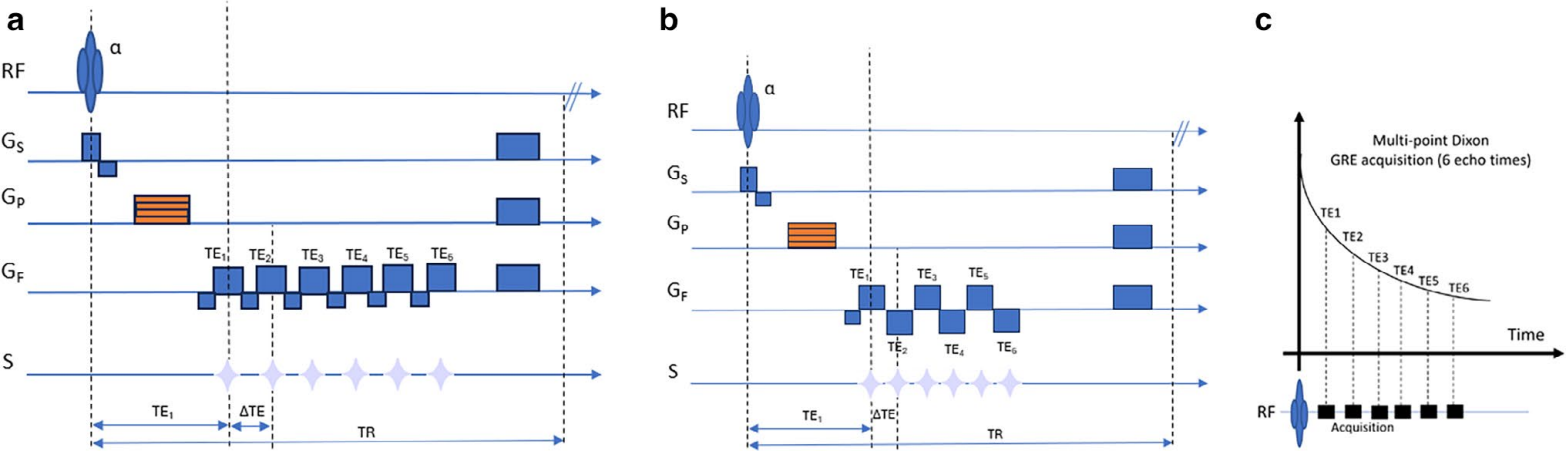
(a) Timing diagram of a multi‐echo gradient recalled echo (GRE) based sequence for fat quantification with unipolar readout (b) Multi‐echo GRE sequence with bipolar readout gradients (c) Multi‐echo Dixon images are used as source images to reconstruct fat and water images as well as to estimate the R2* map. PDFF (fat fraction map) is calculated by using water and fat images, after correcting for R2* decay. RF: Radiofrequency, Gs: Slice gradient, G_P_: Phase gradient, G_F_: Frequency gradient, S: Signal, TE: Echo time, TR: Repetition time.

**FIGURE 4:**
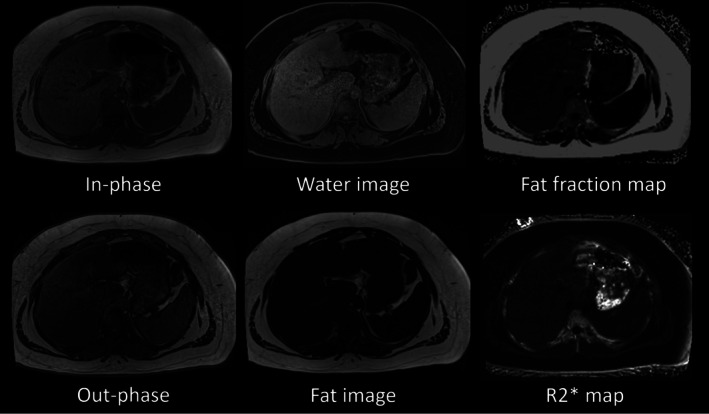
Images of a 16‐year‐old boy with Gaucher disease. Liver fat from the fat fraction map was measured to be 20%.

Most MRI vendors provide FDA‐approved packages (such as IDEAL‐IQ by GE HealthCare; Liver Lab by Siemens Healthineers; mDIXON Quant by Philips Healthcare; Fat fraction quantification by Canon Medical Systems) that can generate PDFF maps, making this technology relatively accessible in clinical settings [18, 21, 38]. On currently available commercial systems, a breath‐hold acquisition is required for accurate PDFF because they employ cartesian‐based reconstruction trajectories that are sensitive to phase errors caused by motion. A six‐echo 3D gradient recalled echo‐based acquisition to estimate PDFF and R2* maps can typically be acquired on most vendor platforms in a single breath‐hold of around 15 s (Table 1). In addition to in‐phase, opposed‐phase, water, and fat images available from conventional imaging, PDFF and R2* quantification images can also be generated from a single acquisition (Figures 3 and 4). A multi‐vendor, multi‐site study using a quantitative fat fraction phantom confirmed that MRI‐PDFF is accurate and reproducible across different sites, vendors, field strengths, and protocols [39]. Furthermore, a meta‐analysis reported that MRI‐PDFF shows excellent linearity, bias, and precision across various vendors, field strengths, and reconstruction methods [17].

**TABLE 1:** Recommended liver fat MRI acquisition parameters.

Parameters	Values
Field strength	1.5T, 3T
Pulse sequence	3D GRE multi‐echo Dixon
Matrix size	160 × 140
NSA	1
First TE (msec)	1
Delta TE (msec)	0.7
No. of echoes	6
TR (msec)	
Flip angle (degrees)	5 or less
FOV (mm)	300–450
Slice thickness (mm)	6
Acceleration factor	2
Acquisition time (min: sec)	0:12 to 0:20

*Note*: Anterior abdomen and posterior spin coils are recommended to be used for all imaging platforms.

Abbreviations: 3D: three‐dimensional, FOV: field of view, GRE: gradient recalled echo, min:sec: minutes: seconds, mm: millimeters, msec: milliseconds, NSA: number of signals acquired, TE: echo time, TR: repetition time.

Schneider et al. found that, despite relatively short breath‐holds (11 to 22 s), 32 out of 438 PDFF series had subcutaneous fat signals overlapping both the abdomen and phantoms, which could lead to artificially high fat percentages due to respiratory motion [38]. Cartesian‐based methods can suffer from motion‐induced coherent aliasing artifacts, which degrade image quality and affect fat quantification accuracy. Infants and young children are particularly prone to more frequent voluntary and involuntary motion and may have difficulty following scan instructions. Techniques like parallel imaging and compressed sensing, which are designed to accelerate MRI acquisition, may also introduce ghosting artifacts, often due to variations in patient positioning between PDFF image acquisition and reference scans. Motion‐compensation strategies, such as navigator gating, have demonstrated nearly perfect agreement in PDFF values compared to standard breath‐hold sequences, offering a reliable alternative for motion‐prone patients [40]. Non‐cartesian radial‐based trajectories are now being made available as a potential solution to overcome these limitations in pediatrics [41, 42]. Zhong et al. conducted a comparison between the 3D free‐breathing stack‐of‐radial technique and the reference 3D breath‐holding Cartesian CSE MRI technique in 16 pediatric patients referred for MRI examinations, showing that stack‐of‐radial MRI with self‐gating motion compensation seems to allow free‐breathing liver PDFF quantification in children [43].

### 4.4 Normal and Recommended Pathologic Cutoff Values

Using metabolic indices as a reference and histology as the standard, on the same lines of adult values, pediatric studies also use a cutoff of 6% fat fraction [44, 45]. The values are interchangeable between 1.5T and 3T sequence acquisitions [45]. The commonly used PDFF thresholds are categorized as follows: less than 6% indicates normal levels, 6%–17% represents mild steatosis, 17%–22% corresponds to moderate steatosis, and greater than 22% indicates severe steatosis [46, 47, 48, 49]. A suggested MRI liver fat reporting template is presented in Data S1.

### 4.5 Limitations in Liver Fat Quantification Using MRI

Despite its superior diagnostic performance, MRI‐based fat determination has some limitations. Areas affected by motion and parallel image artifacts can compromise measurement accuracy, requiring careful region of interest placement to avoid these regions. Additionally, current MRI techniques for R2* correction are limited in patients with high iron levels, as extreme iron overload can cause rapid signal loss, making it challenging to measure accurately. MRI is also less feasible for children under 8 years old, who often require sedation or anesthesia due to difficulty remaining still.

Beyond challenges with fat fraction analysis, MRI's effectiveness can be influenced by patient‐specific factors, operator expertise, and institutional resources. While new MRI platforms offer software packages for processing PDFF maps, some imaging centers may lack these tools due to budgetary and hardware constraints. Patient factors such as claustrophobia, implanted devices, and discomfort can also limit MRI suitability. Furthermore, MRI generally incurs higher costs compared to ultrasound, highlighting a need for future research and development to address these cost concerns.

## 5 Liver Fat Quantification by Ultrasound

**FIGURE 5:**
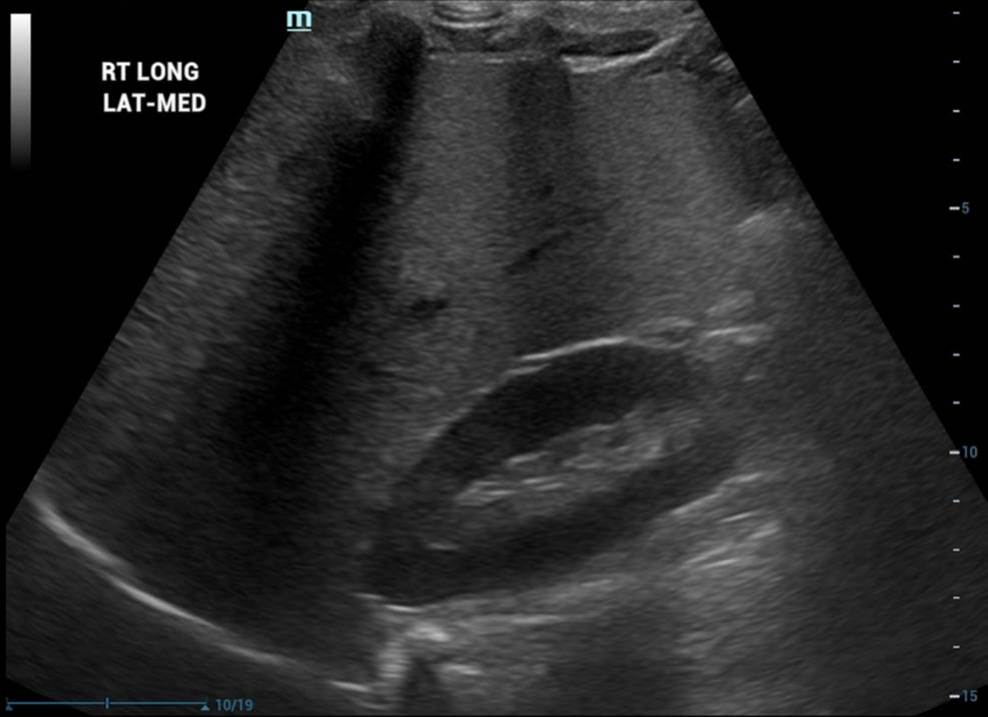
Longitudinal gray‐scale ultrasound image of a 10‐year‐old boy shows echogenic, homogeneous appearance of the liver, consistent with hepatic steatosis. The liver parenchyma is brighter than the cortex of the right kidney.

**FIGURE 6:**
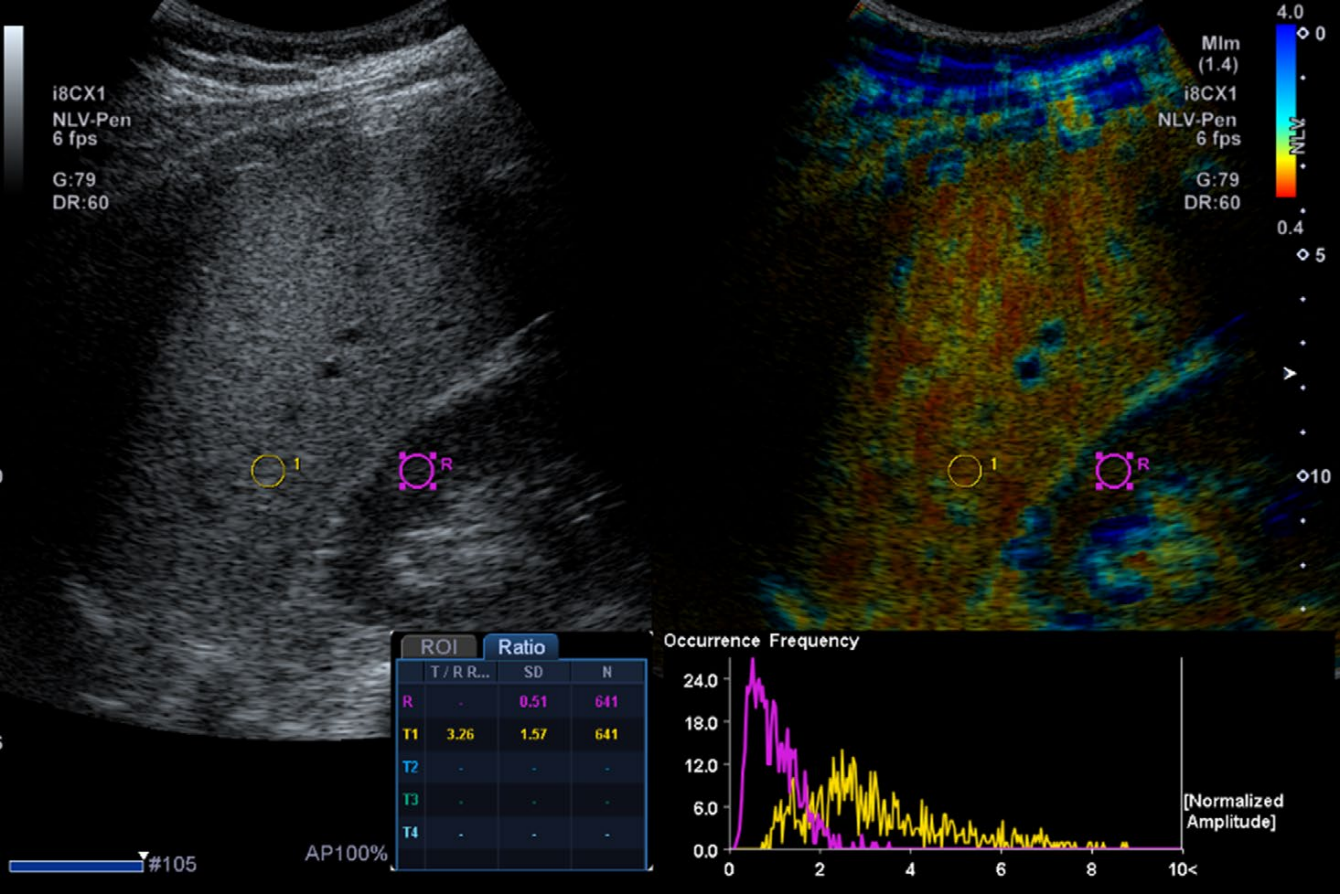
Longitudinal gray‐scale ultrasound image of a 13‐year‐old boy on the left shows echogenic, homogeneous appearance of the liver, consistent with hepatic steatosis. Left gray‐scale image and right color images show placement of small regions of interest in the liver and right kidney parenchyma for automated hepatorenal index calculation.

Historically, the assessment of hepatic steatosis in clinical practice using B‐mode ultrasound has primarily relied on observing increased liver echogenicity, often compared to the echogenicity of the right kidney cortex, along with reduced visibility of the portal triads [50, 51]. In hepatic steatosis, the liver has a diffusely bright appearance with smooth and tightly packed echoes [52]. Semi‐quantitative comparison of liver to kidney echogenicity is referred to as the hepatorenal index (HRI) and has been explored in both children and adults (Figure 5) [53, 54]. The HRI is a simple and low‐cost method for assessing hepatic steatosis, but it is reliant on normal renal echogenicity (i.e., a healthy right kidney) and seems to vary by ultrasound scanner manufacturer based on the literature. The HRI is based on the premise that normal liver parenchyma is similar to or only slightly more echogenic (i.e., “brighter”) than the adjacent right kidney, and that increased values are indicative of hepatic steatosis. Figure 6 shows automated HRI measurement that is available on some US scanners [55]. Automated methods have the potential to improve operator workflow by suggesting placement of ROIs and on‐system HRI calculation. Acoustic wave reflection, scattering, and attenuation by lipid droplets in the setting of liver steatosis cause increased signal to return to the transducer [56] as well as decreased tissue penetration of sound energy. Increased attenuation of the sound due to steatosis can lead to poor visualization of the diaphragm as well as normal structures and lesions in the liver [56].

In a study of 48 children undergoing both MRI‐PDFF and HRI, D'Hondt et al. showed a positive correlation between measurements (*r* = 0.84) [57]. Frankland et al. also showed a positive correlation between MRI‐PDFF and HRI (*r* = 0.51–0.61) in another pediatric cohort (*n* = 69) [23]. However, in a cohort of 44 children and young adults (≤ 21 years of age), Alves et al. demonstrated a considerably weaker correlation between MRI‐PDFF and HRI (*r* = 0.27) [58].

**FIGURE 7:**
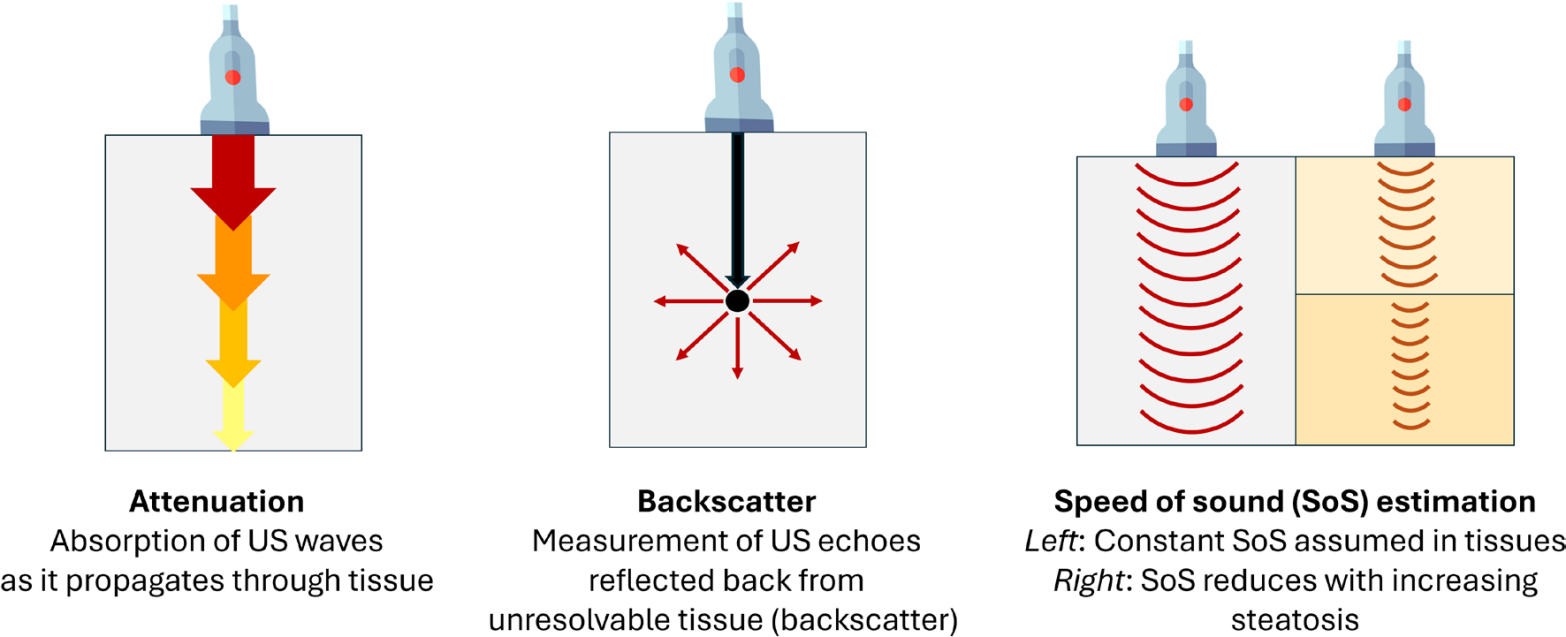
Schematics for the principle behind attenuation, backscatter coefficient, and speed of sound estimation.

Recently, newly developed quantitative ultrasound methods have used approaches based on attenuation of sound, backscattering of sound, and speed of sound estimation (Figure 7) specifically aimed at the accurate quantification of hepatic steatosis. The following section will review several of these ultrasound‐based fat quantification methods.

## 6 Ultrasound Liver Fat Quantification Methods

### 6.1 Controlled Attenuation Parameter

The controlled attenuation parameter (CAP) integrated within vibration‐controlled transient elastography devices (FibroScan; Echosens) was the first non‐invasive quantitative tool developed for assessing hepatic steatosis. CAP measurements are also based on the attenuation of sound waves and are generally acquired in the point‐of‐care setting (e.g., the hepatology clinic) [57, 59]. CAP measurement is performed simultaneously while measuring liver stiffness by transient elastography. While this technology is ultrasound‐based, it is one‐dimensional, and correlative gray‐scale anatomic imaging is not acquired by this method. A recent meta‐analysis by Jia et al. comparing the diagnostic performance of MRI‐PDFF and CAP for diagnosing MASLD in children and adolescents concluded that both techniques can grade hepatic steatosis with a high degree of accuracy [60]. In this study, MRI‐PDFF accurately diagnosed hepatic steatosis with a summary sensitivity of 95% and specificity of 92% compared to 86% and 88%, respectively, for CAP. Interestingly, in a small single‐center study evaluating children with known or suspected MASLD by Alves et al., there was no significant correlation between MRI‐PDFF and CAP [61]. Additionally, it has previously been reported in adult populations that the accuracy of CAP decreases with increasing grades of steatosis [62]. Prior studies have revealed the vast variation between cut‐off values for the detection of hepatic steatosis > 5% [63, 64]. For instance, studies have reported cut‐off values for the detection of steatosis > 5% ranging from 219 dB/m in a cohort of adult patients with viral hepatitis to 302 dB/m in a cohort of adults with suspected MASLD [65, 66]. Although limited in number, pediatric cohorts have also shown similar trends in cut‐off values [67]. This is significant because the etiology of liver disease, BMI, and metabolic factors are all potential confounders and may have an impact on the measured CAP value [68].

### 6.2 Attenuation Coefficient Imaging

**FIGURE 8:**
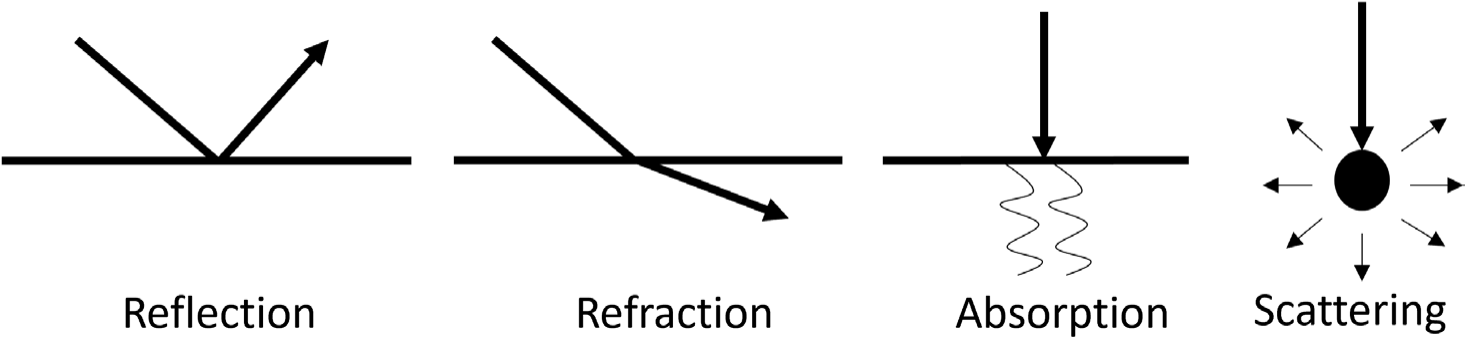
Schematic of ultrasound wave pattern showing reflection, refraction, absorption, and scattering.

During their propagation, acoustic waves are modified due to their interaction with the physical medium. Their direction and amplitude may change due to (a) reflection or (b) refraction at the interface of media with different acoustic impedance. The amplitude may also decrease or attenuate due to (c) absorption and (d) scattering produced by the insonified medium (Figure 8). As mentioned above, the attenuation of sound energy can be qualitatively appreciated by ultrasound providers using standard B‐mode imaging. The attenuation coefficient, which increases with increasing liver triglyceride content, is defined as the ratio of one radiofrequency echo magnitude to another at a different depth, expressed in decibel per centimeter per megahertz (dB/cm‐MHz). The magnitude of loss (A(z)) equals A0—αfz, where A(z) is the amplitude in decibels (dB) after the wave propagates a distance z (in cm), A0 is the initial ultrasonic wave amplitude in dB, α is the attenuation coefficient (in dB/cm‐MHz), and f is the ultrasonic frequency in MHz. By assuming a linear dependency with frequency and calculating slope, the relationship between attenuation and frequency can be determined. Thus, it is common to report attenuation measurements using the term “attenuation coefficient slope” (ACS).

**FIGURE 9:**
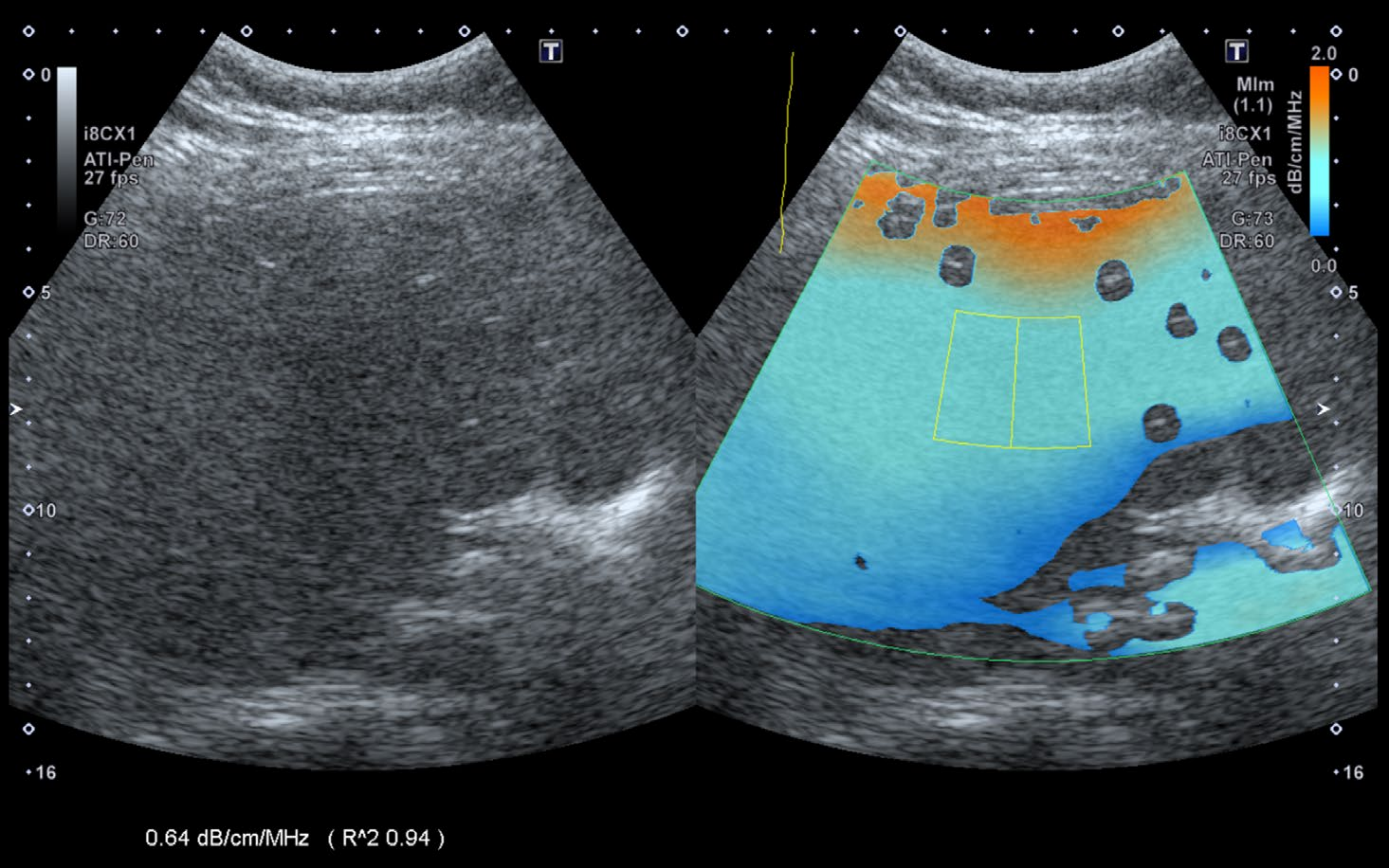
Side‐by‐side gray‐scale and attenuation images of a 17‐year‐old boy show placement of a region of interest in the central liver. The attenuation coefficient was measured as 0.64 dB/cm/MHz. Orange at the top of the color overlay and dark blue at the bottom of the color overlay represent areas of artifact.

Most commercial ultrasound vendors now have a commercially available attenuation coefficient‐based imaging method with different names, including ultrasound‐guided attenuation parameter (UGAP, GE Healthcare), attenuation imaging (ATI, Canon Medical Systems), attenuation measurement (ATT, Fujifilm), and tissue attenuation imaging (TAI, Samsung Medison). These solutions, similar to shear wave elastography technology, typically provide a quantitative measurement along with an image/parametric map [69]. Historically, for this method to achieve accuracy, a calibration step has typically been necessary to account for the transducer's compression wave diffraction, which also diminishes echo magnitude with depth [70]. Calibration is performed by acquiring echo signals from a reference phantom with known attenuation using the same equipment and system settings as the clinical examination [71]. Recently, most manufacturers have adopted embedded calibration techniques based either on training datasets or preset reference phantom measurements (Figure 9) [72, 73]. Attenuation measures are also adjusted for gain and other system factors. A recent study in adults by Ferraioli et al. suggests that attenuation coefficient measurements in the liver are dependent on both depth below the liver capsule and region of interest size [74].

In a study of 48 children undergoing both MRI‐PDFF and ultrasound attenuation coefficient imaging, D'Hondt et al. showed a positive correlation between measurements (*r* = 0.76) [57]. Alves et al. also demonstrated a moderate positive correlation between MRI‐PDFF and attenuation coefficient imaging in a cohort of 44 children and young adults (≤ 21 years of age) (*r* = 0.45) [61]. Dardanelli et al. performed attenuation coefficient imaging in 174 children without any technical failures and with excellent inter‐operator agreement (ICC = 0.94) [75]. Another study evaluated the measurement of the UGAP (GE Healthcare) in 118 children using a high‐frequency (2–9 MHz) convex transducer. This study found an attenuation coefficient of 0.699 dB/cm‐MHz to be 90% sensitive and 100% specific in the detection of hepatic steatosis in children [76].

Previous work has shown that the attenuation coefficient in the liver can be in the range of 0.43–1.26 dB/cm‐MHz across varying degrees of steatosis [75]. However, the different vendors with attenuation measurement algorithms define varied cut‐off values for the detection of > S0 steatosis. For example, cut‐off values for detecting > S0 steatosis vary among different ultrasound attenuation measurement algorithms: UGAP (> 0.65), ATI (> 0.67), and ATT (> 0.62) [77]. Such differences in threshold values are due to variation in system calibration with different phantoms/datasets, algorithms, and patient populations. A meta‐analysis done on prospective studies on the attenuation coefficient showed that the median attenuation coefficient value required to detect steatosis > S0 is 0.63 dB/cm‐MHz [78]. Similar results have been shown in pediatric populations through prospective studies. Polti et al. showed TAI (Samsung Medison) to be 100% sensitive and 86% specific (AUROC 0.903) in detecting hepatic steatosis when compared to MRI‐PDFF [79]. In a prospective study of 86 children, the median attenuation coefficient measured by ATI (Canon Medical Systems) was 0.65 dB/cm‐MHz in healthy, normal‐weight children [80]. The authors also found that the attenuation values were slightly higher compared to adults, although no significant correlation with age was found upon analysis [80].

A suggested ultrasound attenuation coefficient imaging reporting template is presented in Data S2.

### 6.3 Backscatter Coefficient Imaging

Backscatter coefficient (BSC) imaging involves analyzing the echoes received by the ultrasound transducer, which result from the reflection and scattering of compression waves [70]. These echoes are modulated in magnitude by the constructive and destructive wave interferences caused by the tissue microstructure [70]. A requirement for BSC measurement is to obtain a reference BSC in cm^−1^ sr^−1^ at a specific frequency or at different frequencies within the bandwidth of the ultrasound transducer to compare the RF signal from the insonified organ to that of the reference for calibration. For clinical applications, most ultrasound manufacturers now include built‐in calibration using preset reference phantom measurements to account for system effects [73]. Only a limited number of studies have studied the efficacy of BSC as a quantitative measure of hepatic steatosis. In an adult study of 204 patients with and without MASLD, Lin et al. showed that BSC measurements can accurately diagnose (sensitivity = 87%, specificity = 91%) and quantify hepatic steatosis using MRI‐PDFF as the reference standard [81]. Another study revealed a positive correlation between BSC and MRI‐PDFF ≥ 5% (Pearson correlation coefficient of 0.58) [82]. Although a few studies on BSC have shown good results in adults [70, 74, 75], the stand‐alone technique of BSC measurement for the detection of hepatic steatosis in pediatric populations has not been studied.

Measured at 3 MHz, prior work has shown normal livers to have a BSC of 0.0003–0.0007 cm^−1^ sr^−1^ [83], whereas steatotic livers display a BSC of 0.0031–0.0105 cm^−1^ sr^−1^ [84]. Measurement of BSC requires radiofrequency (raw) data and precise consideration of the system settings and beam properties [85].

### 6.4 Speed of Sound Imaging

For purposes of image production as they convert time to distance, most ultrasound scanners assume a constant speed of sound of compression waves of 1540 m/s. In reality, the actual speed of sound waves varies depending on tissue structural characteristics, including density and bulk modulus [52]. Changing tissue composition produces errors as the real speed of sound in soft tissues varies by ±150 m/s, or about 10% of the assumed speed of sound. The tissue‐dependent variations in the speed of sound can be retrieved and used as a potential biomarker providing acoustic impedance contrast [86]. The speed of sound changes with tissue histologic changes and is lower in fat‐containing tissues, including hepatic steatosis (the decrease in speed of sound is correlated with an increased amount of liver fat) [87, 88, 89]. The literature on speed of sound estimation in adults is scarce, as is speed of sound data in children. To our knowledge, only one study has reported findings related to speed of sound estimation in a pediatric population. Born et al. evaluated a population of 75 children and adolescents, comparing speed of sound estimates of hepatic steatosis with MRI‐PDFF and showed that liver speed of sound correlates with body mass index and is lower in overweight and obese individuals [90]. The paucity of literature on the speed of sound estimation for hepatic steatosis is at least in part due to the limited availability of such an algorithm on few commercially available clinical systems. Additionally, S1–S3 stages of hepatic steatosis exist within a narrow range of speed of sound, requiring highly precise measurements to accurately detect and/or grade hepatic steatosis.

### 6.5 Multiparametric Approaches Toward Hepatic Fat Quantification

**FIGURE 10:**
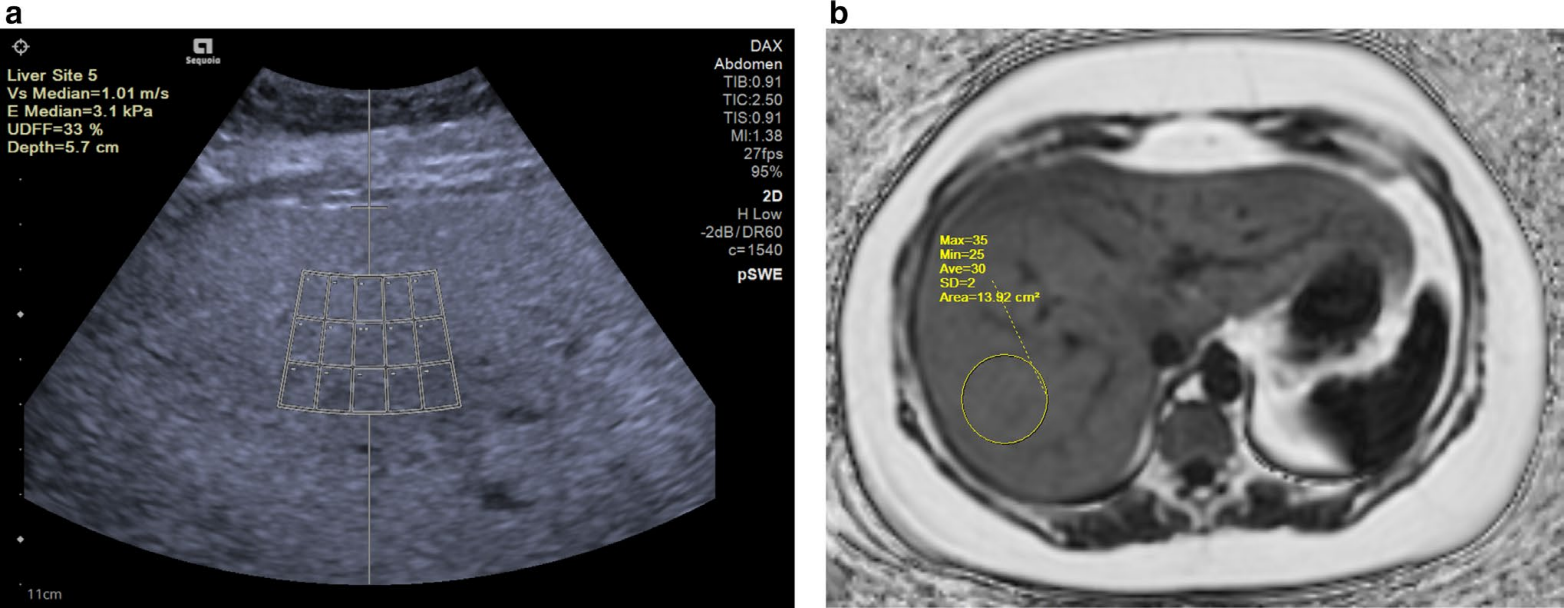
(a) Ultrasound‐derived fat fraction (UDFF) image of a 16‐year‐old girl shows that the liver is diffusely echogenic. The UDFF measured 33%. (b) Axial PDFF MR image of the same patient measures 30% in the posterior right lobe.

Multiparametric ultrasonographic fat quantification combines multiple independent sonographic parameters to adjust for the various confounders that may affect the true fat quantification value. UDFF is a recently FDA‐cleared multiparametric method for measuring hepatic steatosis that incorporates both the attenuation coefficient and BSC measurements [73]. UDFF is available on a single ultrasound system and produces a UDFF value expressed as a percentage, scaled to match MRI‐PDFF (Figure 10). This method adjusts for system effects and some confounders through the integration of reference phantom measurements [73]. Recently, Dillman et al. reported that UDFF strongly correlates with MRI‐PDFF measurements and provides good diagnostic performance for detecting hepatic steatosis in overweight and obese adolescents and adults (age ≥ 16 years) [22]. Zalcman et al. also demonstrated a positive correlation between MRI‐PDFF and UDFF (ICC = 0.92) in a sample of 46 pediatric patients [91].

### 6.6 Acoustic Structure Quantification

**FIGURE 11:**
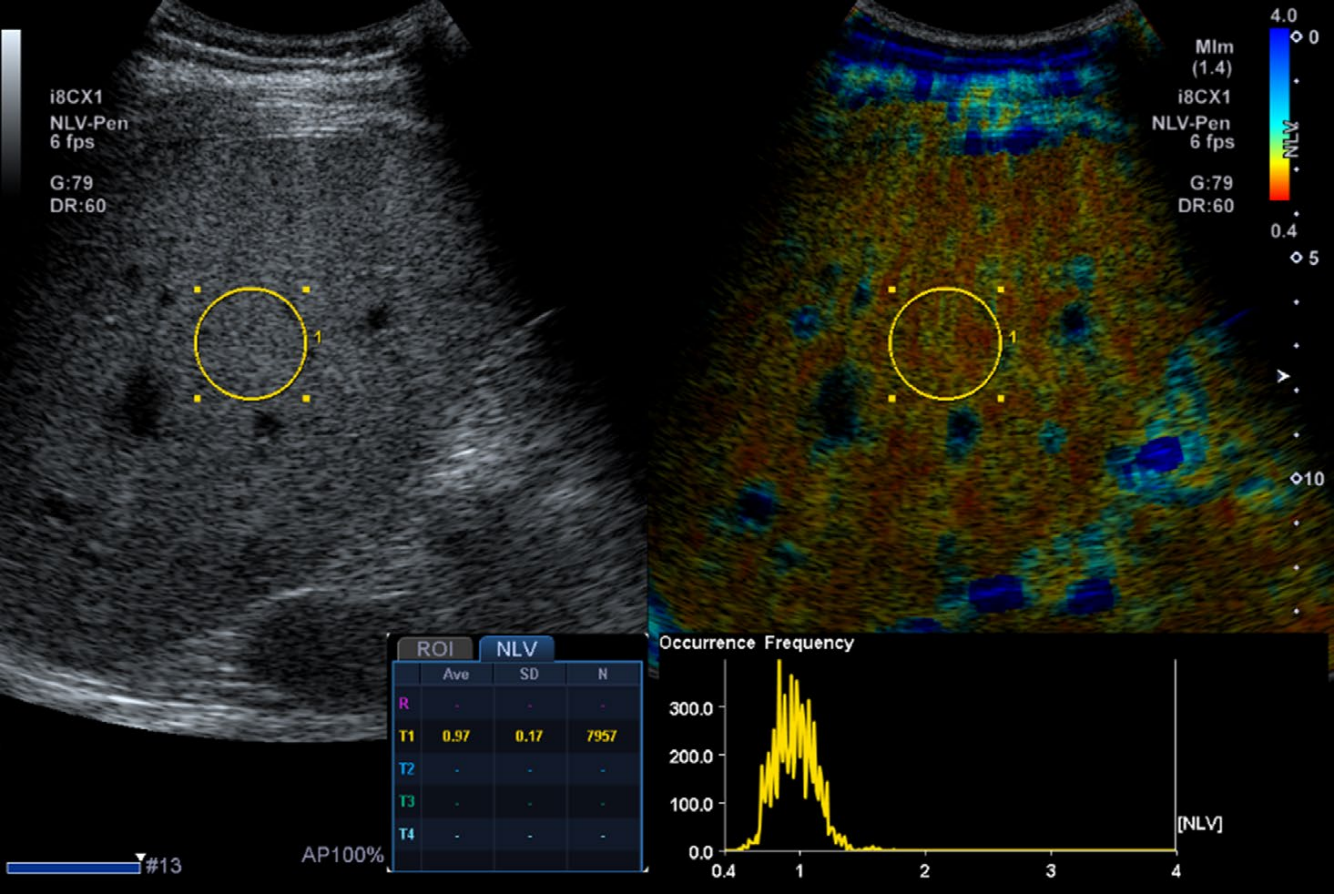
Side‐by‐side gray‐scale and normalized local variance (NLV) images of a 13‐year‐old boy show placement of a region of interest in the central liver. NLV evaluates the distribution of echo amplitudes to assess for tissue structural changes.

Acoustic structure quantification (ASQ), including normalized local variance (NLV), is a recently developed quantitative analysis method for assessing hepatic steatosis that is available on a single ultrasound system (Figure 11) [92, 93, 94]. ASQ compares theoretical and actual echo amplitude distributions. The theoretical distribution is modeled using the Rayleigh distribution function, based on the assumption that speckle patterns arise from the interference of ultrasound beams interacting with very small scattering objects smaller than the ultrasound beam wavelength. By comparing the theoretical distribution with the real distribution, ASQ provides quantitative insights into changes in parenchymal echotexture associated with diffuse liver diseases, such as steatosis [95]. As hepatic steatosis progresses, the liver's small structures, including small vessels, become blurred, leading to increased echogenicity and a more homogeneous appearance of the liver parenchyma. The focal disturbance (FD) ratio calculated using ASQ offers quantitative information on these echotexture changes. Lee et al. demonstrated a significant positive correlation between the FD ratio from ASQ and the hepatic fat fraction estimated by MR spectroscopy [92]. Although promising, studies on ASQ and NLV are limited in number, small sample sizes, lack of adjustment for confounders, and lack of generalizability. Pediatric data is also lacking.

### 6.7 Technical Considerations in US‐Based Fat Quantification in Children

Certain technical considerations should be made when comparing ultrasound exam performance of the liver between adults and children (Table 2). Firstly, adults generally have a relatively higher amount of subcutaneous fat than children, which can attenuate ultrasound waves as they pass through tissue, decreasing the clarity of the image produced. This requires the use of a lower frequency transducer (e.g., 2–5 MHz) to optimize depth penetration. In contrast, higher‐frequency probes (e.g., 5–10 MHz) can potentially be used in children, thus providing better resolution [76]. Additionally, finding the optimal scanning window via intercostal space can be challenging in adults due to larger rib sizes and requires careful and optimal positioning of the patient and transducer. In contrast, the wider and more flexible intercostal spaces in the pediatric population (owing to smaller rib size and less muscle mass) allow for a less challenging window access to scan the liver [97].

**TABLE 2:** Suggested generalized acquisition protocol and requirements for attenuation estimation across vendors.

Protocol	Recommendations
Food intake status	Fasting is not mandatory if *only* attenuation measurement is being acquired
Breath‐hold	Breath‐hold at exhale is recommended
Ultrasound mode	Best quality of the B‐mode image, but not necessarily with the transducer perpendicular to the liver capsule
Liver probe positioning (external)	Right intercostal space
Liver measurement positioning (internal)	Measurement box should be positioned perpendicular to the transducer
Measurement box size	Length of the measurement box 3 cm
Measurement box depth	Upper edge of the measurement box at 2 cm below the liver capsule
Measurement box range	IQR/M ≤ 15%
No. of acquisitions	Median (or mean) value of three to five acquisitions
Measurement reliability	For reliable measurements, vendor specific thresholds and quality control criteria should be applied

*Note*: Adapted from the World Federation of Ultrasound in Medicine and Biology guidance on liver fat quantification by Ferraioli et al. [96] with note that this has not been validated in pediatric patients.

Abbreviation: IQR/M, interquartile range/median.

Although adult patients are generally compliant and able to follow instructions to perform breath‐holding or change positions to optimize the scanning window, pediatric patients, especially infants and younger children, may not be able to cooperate as easily. Thus, faster scanning techniques, distractions (e.g., toys and videos), and, in rare cases, sedation may be needed to obtain optimal images. In this case, MRI should be considered over ultrasound, as its diagnostic performance and accuracy when high‐cost clinical resources are to be utilized.

Measuring hepatic steatosis by ultrasound offers numerous challenges. Most quantitative imaging methods rely on computations over windows that are generally 5 to 10 times larger than the point spread function, which reduces spatial resolution compared to B‐mode imaging. Although spatial filtering can help mitigate this window effect, mathematical regularization is another technique that has been used. Regularization not only addresses the window effect but also helps reduce outlier values that can appear as background noise on images. Work also continues to develop phantom‐free methods for detecting and measuring liver fat to improve the clinical workflow, although validation of such methods remains critical.

### 6.8 Reporting Considerations in Ultrasound‐Based Hepatic Fat Quantification

To date, all ultrasound quantitative methods for measuring hepatic steatosis use vendor‐specific algorithms, and there is a general paucity of published high‐quality normative data, particularly in the pediatric population. While attempts at standardization via various organizations such as the American Institute of Ultrasound in Medicine (AIUM) and the World Federation for Ultrasound in Medicine and Biology (WFUMB) are in progress, especially towards acquisition protocol standardization [77, 96], more studies are needed to evaluate ideal cut‐off values in the pediatric population. In the interim, we recommend that vendor‐specific cut‐off values for distinguishing normal from steatotic liver should be used prior to larger validation cut‐off for grading hepatic steatosis in the pediatric population. Appropriately designed pediatric studies are needed for all of the above techniques in healthy participants to understand what range of values is normal. Until ultrasound techniques are standardized among vendors, these studies need to be performed for each vendor deploying a particular method. Furthermore, same‐day and between‐day repeatability as well as between‐system reproducibility must be studied in the pediatric population for robust validation, as has recently been done in the adult population [98].

Similar to the evolution of ultrasound shear wave elastography acquisition parameters, the exact technique used to measure hepatic steatosis, including the number of measurements obtained and ultrasound system and transducer, needs robust validation. For now, the WFUMB guidelines have recommended an acquisition protocol in the adult population and can be extrapolated to the pediatric population [96].

### 6.9 Limitations in Liver Fat Quantification Using Ultrasound

Conventional ultrasound is subject to operator variability, often leading to inconsistent results. Patient‐related factors, such as body habitus impacting the subcutaneous thickness that also attenuates sound waves, can also affect the diagnostic performance of ultrasound methods. Technical failure is more common in patients with a larger body habitus, where MRI offers an advantage over ultrasound. Given that ultrasound measurements provide indirect estimates of liver fat as an extrapolation of the technique used, making them dependent on calibration and acquisition parameters. This dependency can result in variable measurements over time and across different manufacturers, machines, and operators, potentially impacting patient care. Additional studies are needed to understand how other liver histologic alterations (e.g., fibrosis) impact these quantitative ultrasound measurements. Additionally, liver steatosis may be diffuse, focal, or heterogeneous in distribution, limiting the ability of ultrasound to capture these, given the protocols usually allow an intercostal approach and acquisitions from suitable acoustic windows of the liver. Thus, in the appropriate clinical context, MRI should be considered for validation.

## 7 Recommendations for Clinical Care

MRI‐PDFF is a precise, reproducible, and repeatable method for quantifying hepatic steatosis, effectively measuring fat content in the liver [17, 18, 99]. This technique also provides R2* maps, which, along with the built‐in PDFF correction, is useful for concurrently assessing iron deposition in the liver [18, 100].

Advanced quantitative MRI techniques offer several advantages over ultrasound [21]. MRI‐PDFF, a fundamental tissue property, does not require internal calibration or reference standards. MRI sequences can account for biological confounders, such as iron overload, by simultaneously measuring and correcting for R2* [16, 100]. Additionally, MRI can acquire the images needed for PDFF measurements very quickly, often capturing the entire liver in a single breath‐hold [21]. This allows for volumetric assessment of hepatic steatosis, a capability not yet available with ultrasound. Furthermore, MRI tends to be less dependent on operator skill and patient factors compared to ultrasound.

Clinical care recommendations also depend on imaging test availability, local radiologist expertise, and patient factors. If a highly accurate measurement of liver fat is needed (e.g., if required to gain access to a novel medication or a clinical trial), then MRI‐PDFF should generally be the method of choice. However, if screening of large cohorts is desired, particularly at lower costs, ultrasound may be the more cost‐effective method. Although these are not as well validated as MRI‐PDFF, they can serve as screening modalities to select patients that may warrant an MRI evaluation. Overall, quantitative ultrasound methods are preferred in children with a contraindication to MRI or when sedation or anesthesia is needed. Fortunately, both MRI and ultrasound allow rapid measurement of liver fat, with currently available MRI sequences requiring less than 15–20 s to image the entire liver and ultrasound requiring less than 2 min to obtain three to five measurements (Table 3).

**TABLE 3:** Summary of MRI and Ultrasound based fat quantification methods.

Technique	Advantages	Disadvantages	Notes
MR based liver fat quantification methods			
MRS	Considered the most accurate method for measuring hepatic fat content at user‐specified locations within the liver.	Limited by sampling bias.	
PDFF	The most advanced and fully quantitative approach aimed at accurate and precise measurement of steatosis.	A relatively new method and may not yet have widespread availability.	
Ultrasound based liver fat quantification methods			
CAP	Easy to use at point of care in hepatology clinics. Multiple validation studies.	One dimensional calculation without imaging support. Varying accuracy.	The first non‐invasive quantitative tool developed for assessing hepatic steatosis.
AC	Measures the energy loss of ultrasound waves as they pass through the liver, correlating with fat content. Most common, clinically available approach used by several vendors. Provides a continuous, quantitative parameter for fat assessment.	Depth‐dependent variability can influence reliability. Significant variability between vendors.	Factors such as patient habitus (e.g., increased subcutaneous thickness) and overlapping effects from concurrent fibrosis can impact the value.
BSC	Measures the scattering strength of liver tissue, which changes with varying degrees of fat accumulation. Provides a more direct measure of hepatic microstructure compared to AC.	Like AC, can be impacted by confounding factors such as subcutaneous tissue thickness. Values are challenging to report with unfamiliar unit of measurement.	BSC measurement alone is not a commercially available technique. BSC requires sophisticated calibration techniques to improve accuracy.
SSE	Directly measures the speed of sound waves traversing through liver tissue, which changes with varying fat content. Subcutaneous impact can be accounted for in different approaches. Less influenced by depth‐related factors.	Limited vendors use this approach and needs robust validation. May be heavily dependent on accurate reference speed measurements.	Has a narrow range of measurement, thus, requires high level of precision for accurate measurement of fat content.
UDFF	Combines attenuation and backscatter measurements, providing a more comprehensive assessment of hepatic fat than either parameter alone. UDFF values can be expressed as a percentage, scaled to match MRI‐PDFF.	Reported significant variability between vendors.	

Abbreviations: AC: attenuation coefficient, BC: backscatter coefficient, CAP: controlled attenuation parameter, MRS: magnetic resonance spectroscopy, PDFF: proton density fat fraction, SSE: speed of sound estimation, UDFF: ultrasound derived fat fraction.

### 7.1 Artificial Intelligence Applications in Liver Fat Quantification

Recent advancements in artificial intelligence (AI) and deep learning (DL) models have shown promise in improving both MRI and ultrasound techniques for diagnosing hepatic steatosis and predicting an estimated liver fat fraction. AI models can process standard in‐phase and out‐of‐phase MRI data to automate liver segmentation, identify regions of interest, compute fat fraction, and correct for potential confounders such as iron overload or motion artifacts [101]. These models facilitate the adoption of PDFF, expand opportunities and diagnostic accuracy, streamline workflows, and reduce operator dependency. In a recent study, the authors aimed to develop AI algorithms to detect hepatic steatosis from gray‐scale ultrasound images (MRI‐PDFF threshold of over 6.4), achieving a sensitivity of 72%, specificity of 95%, and diagnostic accuracy of 83% [102]. Another study evaluated two deep learning models using RF data to diagnose MASLD and estimate fat fraction, reporting an AUC of 0.98, sensitivity of 97%, specificity of 94%, and accuracy of 96% for diagnosing MASLD [103]. These studies highlight the significant potential of AI and DL algorithms in improving the accuracy and automation of liver fat detection and quantification, suggesting further research could lead to automated, quantitative, and cost‐effective evaluation of hepatic steatosis.

## 8 Conclusion

In conclusion, MRI and ultrasound have emerged as critical tools for non‐invasive liver fat assessment in children, each offering unique advantages tailored to specific clinical needs. MRI‐PDFF is relatively more established and is ideal for quantification and longitudinal monitoring of hepatic steatosis. Meanwhile, advancements in ultrasound technologies, such as UDFF and attenuation coefficient imaging, offer cost‐effective and accessible screening options. These complementary approaches can help improve early detection, minimize invasive procedures, and guide therapeutic interventions for pediatric MASLD. Ongoing standardization, validation studies, and the integration of AI will further enhance the utility of these imaging modalities, optimizing pediatric liver care.

## Supporting information


**Data S1.** Supporting Information.

**Data S2.** Supporting Information.
