# Supplementary material for: MR and Ultrasound for Liver Fat Assessment in Children: Techniques and Supporting Evidence
Source: J Magn Reson Imaging. 2025 Mar 5;62(3):691–706. doi: 10.1002/jmri.29756 (PMC12335346; doi:10.1002/jmri.29756)
Supplement: Supplementary file 1 — Data S1. Supporting Information. [file JMRI-62-691-s002.docx]

HISTORY: Reason

COMPARISON: Comparison

TECHNIQUE: MR examination of the abdomen was performed on a [1.5/ 3.0] Tesla scanner without IV contrast according to the routine metabolic liver and bone marrow protocol. The fat content in the liver has been quantified using [specify method, e.g., Proton Density Fat Fraction (PDFF), MRI spectroscopy, etc.].

No additional sequences were required. No IV contrast was administered.

Volumetric 3-D reconstructions of the liver were created at an independent workstation. These measurements are essential in assessing the severity of disease.

PATIENT EVENTS: None reported.

FINDINGS: In representative areas of the liver, the mean proton density fat-fraction (PDFF) is [ ]% (range [ ] – [ ]%).

Histological steatosis grades by PDFF:

_ <6% = Normal

_ 6 – 17% = Mild

_ 17 – 22% = Moderate

_ >22% = Severe

VISCERAL MORPHOLOGIC:
Liver: No focal hepatic lesion or cirrhotic morphology. The hepatic fat content is consistent with [diagnosis, if applicable, e.g., MASLD]

IMPRESSION:
Fat fraction: fat fraction impression [Normal/Mild/Moderate/Severe].

The liver exhibits [degree of hepatic steatosis], which may require further clinical follow-up based on the patient's history and risk factors. The findings suggest [diagnosis or suggestion for management, if applicable].
