# Supplementary material for: MR and Ultrasound for Liver Fat Assessment in Children: Techniques and Supporting Evidence
Source: J Magn Reson Imaging. 2025 Mar 5;62(3):691–706. doi: 10.1002/jmri.29756 (PMC12335346; doi:10.1002/jmri.29756)
Supplement: Supplementary file 2 — Data S2. Supporting Information. [file JMRI-62-691-s001.docx]

**Supplement 1. Representative reporting template for MRI hepatic fat quantification and assessment**

HISTORY: Reason

COMPARISON: Comparison

TECHNIQUE: MR examination of the abdomen was performed on a [1.5/ 3.0] Tesla scanner without IV contrast according to the routine metabolic liver and bone marrow protocol. The fat content in the liver has been quantified using [specify method, e.g., Proton Density Fat Fraction (PDFF), MRI spectroscopy, etc.].

No additional sequences were required. No IV contrast was administered.

Volumetric 3-D reconstructions of the liver were created at an independent workstation. These measurements are essential in assessing the severity of disease.

PATIENT EVENTS: None reported.

FINDINGS: In representative areas of the liver, the mean proton density fat-fraction (PDFF) is [ ]% (range [ ] – [ ]%).

Histological steatosis grades by PDFF:

_ <6% = Normal

_ 6 – 17% = Mild

_ 17 – 22% = Moderate

_ >22% = Severe

VISCERAL MORPHOLOGIC:
Liver: No focal hepatic lesion or cirrhotic morphology. The hepatic fat content is consistent with [diagnosis, if applicable, e.g., MASLD]

IMPRESSION:
Fat fraction: fat fraction impression [Normal/Mild/Moderate/Severe].

The liver exhibits [degree of hepatic steatosis], which may require further clinical follow-up based on the patient's history and risk factors. The findings suggest [diagnosis or suggestion for management, if applicable].

**Supplement 2. A representative reporting template that may be utilized for ultrasound-based hepatic fat quantification across clinically available techniques. The acquisition protocol and quality criteria followed should be vendor/manufacturer specific.**

**INDICATION**: [*Exam Indication*]

**TECHNIQUE:** [Focused ultrasound evaluation of the liver with fat quantification. Volumetric sweeps were obtained and reviewed.]

**COMPARISON:** [*Comparison study*]

**FINDINGS:**

**LIVER:** [*Blank/Diffusely echogenic liver parenchyma is compatible with steatosis.*] No focal lesions.

**FAT QUANTIFICATION**:

**VENDOR:** [*Vendor name*]

**Fat Quantification approach:** [*Attenuation coefficient*] / [*Speed of sound estimation*] / [*Multiparametric (attenuation + backscatter)*]

**Values:** Using the approach above the *[median/mean]* values are ____ [*dB/cm/MHz*] / [*m/sec*] / [*%*] with a range of [ ] and IQR to median ratio (IQR/M): [ ] %.

**IMPRESSION:**

Fat Quantification:

Which corresponds to the [*presence / absence*] of steatosis.

*****Please include (vendor specific) range of values***

*As example: The following text can be included for a GE vendor* (GE HealthCare. Ultrasound-Guided Attenuation Parameter (UGAP). May 2024. Available from: <https://www.gehealthcare.com/-/jssmedia/GEHC/US/Files/Products/Ultrasound/whitepaper-ugap-giu-logiq-may-2024-jb29271xx.Last?srsltid=AfmBOor48pcKA50bdlSkjo9l3HZiNmr1DD775x2VDq-Z__UUMcT_pN5-&srsltid=AfmBOorZRifrMLHn9GtEmIi2MHEVwkYi4VmOPyXaxE9x9k1RE2A8K8pU>)

[(>30%) *Attenuation IQR/M greater than 30% suggests an unreliable liver steatosis estimate. / (=30%) Attenuation IQR/M of 30% suggests a reliable liver steatosis estimate. / (<30%) Attenuation IQR/M less than 30% suggests a reliable liver steatosis estimate.*]

The median attenuation coefficient is [*attenuation*] dB/cm/MHz, [(<0.65 dB/cm/MHz) *which is unlikely to reflect steatosis. /* (=0.65 dB/cm/MHz) *which suggests steatosis. /* (>0.65 dB/cm/MHz) *which suggests steatosis.]*

Attenuation parameters are based on vendor specific guidelines. Attenuation of 0.65dB/cm/MHz or greater is consistent with steatosis. GE HealthCare. Ultrasound-Guided Attenuation Parameter (UGAP), May 2024
